# Breaking the operator variability in Kibler’s scapular dyskinesis assessment

**DOI:** 10.1007/s12306-024-00834-0

**Published:** 2024-06-21

**Authors:** L. D’Antonio, G. Fiumana, M. Reina, E. Lodi, G. Porcellini

**Affiliations:** 1 Department of Sport Medicine, University of Modena and Reggio Emilia, Modena, Italy; 2 Shoulder Team S.R.L., Forlì, Italy; 3 Department of Orthopaedics and Traumatology, IRCC A. Gemelli University Polyclinic Foundation, Rome, Italy; 4 Centro P.A.S.C.I.A. (Programma Assistenziale Scompenso cardiaco, Cardiopatie dell’Infanzia e A rischio), University of Modena and Reggio Emilia, Modena, Italy; 5 Department of Orthopaedics and Traumatology, University of Modena and Reggio Emilia, Modena, Italy

**Keywords:** Scapula, Kinesis, Scapular dyskinesis, Kibler classification, 3D, Motion analysis

## Abstract

**Introduction:**

Alterations of scapular kinematics are generically reported as scapular dyskinesis (SD), and are a nonspecific response to various shoulder pathologies. The most widely used classification is Kibler’s (K), which is, however, characterized by poor sensitivity. To overcome this limit, using a 3D motion analysis system, we identified a specific pattern for each type of SD according to Kibler.

**Materials and methods:**

We analyzed 34 patients with a total of 68 shoulders who came to our observation for shoulder pain. All patients underwent clinical examination, video-recording and motion analysis with SHoW Motion 3D kinematic tracking system (SM). Three independent observers classified SD into K types I, II and III. Only patients with concordant classification among the 3 operators were studied to identify a characteristic graphic pattern by type of SD.

**Results:**

Typical patterns emerged from the examination with SM. K. type 1 consists of decreased or reversed posterior tilt and increased protraction in flexion–extension (FE) in early degrees of motion. K. type 2 consists of increased protraction and marked reversal of lateral rotation in abduction–adduction (Ab–Ad) in early degrees of movement. K. type 3 has been subdivided into two subgroups: K. type 3-A, composed of patients with massive rotator cuff lesions, shows an increase in all scapular movements in both FE and Ab–Ad. K. type 3-B, composed of patients with scapular stiffness and/or impingement, presents a slight increase in posterior tilt and lateral rotation in the final grades of FE and Ab–Ad.

**Conclusions:**

The SM system allows reproducible dynamic analyses with low intra- and intra- operator variability. In our study, we demonstrated its applicability in the classification of SD. It also provides an objective and quantitative assessment of motor pattern alteration that is essential in the follow-up of patients to evaluate the effectiveness of rehabilitation and/or surgical treatment.

**Level of Evidence 3:**

According to "The Oxford 2011 Levels of Evidence".

## Introduction

Scapular dyskinesis (SD) is defined as abnormal scapular motion and scapulo-humeral rhythm.

Scapular motion consists in 3 rotational movements such as upward/downward rotation around an axis perpendicular to the scapular body, internal/external rotation around a vertical axis and anterior/posterior tilt around an horizontal axis in the plane of the scapula and 2 translation movements involving the clavicle such as retraction/protraction around the thorax and upward/downward translation on the thoracic wall [1–3].

These movements depend on groups of muscle that insert into the scapula and humeral head leading to proper stabilization and thus allow a coordinated movement of the upper arm. Several joints are involved too in the global range of motion of the shoulder, such as: glenohumeral, scapulothoracic, sternoclavicular and acromioclavicular joints. Any modification of one of the above elements can affect the kinematic chain [4]. Therefore, SD is a non-specific response to different problems affecting the shoulder and it correlates with many different pathological conditions [5] that can be divided into proximal (nerve injury, muscle weakness) or distal (rotator cuff injury, acromioclavicular joint injury, gleno-humeral instability, labral tears, impingement syndrome) [6].

Kibler et al. classified SD in three different patterns: displacement of the inferior medial angle of the scapula from the posterior thorax (type I), displacement of the entire medial border of the scapula (type II), early scapular elevation or excessive scapular upward rotation during arm elevation (type III) [7].

It is believed that proper correction of each type of SD covers a pivotal role in shoulder rehabilitation, but scapular evaluation is challenging and many techniques have been used to quantify SD. Given that none of such techniques can ever be performed alone, but always in the context of a broad clinical evaluation, achieving the best possible evaluation of the scapular kinesis is desirable given that proper identification of SD even in asymptomatic athletes may prevent a possible evolution to a symptomatic condition [8].

The purpose of this study is to evaluate SD during active arm elevation using a motion tracking system, the SHoW Motion 3D kinematic system® and classify the observed SD in a Kibler-like pattern.

## Materials and methods

We evaluated 34 patients, with a total of 68 shoulders, who occurred to our observation for painful shoulder. The participants included 10 females and 24 males with an average age of 42.0 years (range 13–74). 6 of the participants were either professional or non-professional but high- level athletes.

All of the participants underwent a clinical examination. Diagnostic investigations had already been performed in most of the patients, including ultrasonography, magnetic resonance imaging (MRI) and CT scan.

The pathologies responsible for the painful shoulder are enumerated in Table 1 and mainly included rotator cuff disease and multiple shoulder instability (mostly posterior).

**Table 1:** Pathologies responsible of shoulder pain, and their incidence among the studied population

Lesion	Number
Shoulder instability	15
Rotator cuff lesion	9
Anatomical shoulder arthroplasty	3
Mumford treatment	2
Neurological lesion	2
Winged scapula	1
Clavicle fracture	1
Postero-superior impingement	1

No exclusion criteria was applied, as our objective was to evaluate SD independently to the cause.

After clinical evaluation, subjects underwent video recording and evaluation with a system that assesses the three-dimensional humero-scapulo-thoracic kinematics using wearable technology in an outpatient setting, the SHoW Motion 3D kinematic tracking system (NCS Lab, Carpi, Italy) [9]. This system includes seven wireless miniature inertial measurement units, each of which provides both raw data (from an accelerometer, a magnetometer and a gyroscope included in the unit) and the orientation matrix. Data is wirelessly sent to a laptop and processed by proprietary software. Said sensors were applied as follows, on the standing subject: two on the lateral aspect of the arms, two in the dorsal aspect of the forearms, two on the spine of the scapulae and one on the manubrium of the sternum. These positions have been validated by the motion analysis protocol named ISEO (INAIL Shoulder and Elbow Outpatient protocol), which presents a high intra- and inter-operator agreement [10].

The anatomical coordinate systems were created by acquiring static reference measurements with the subject standing upright, the humerus positioned alongside the body and the elbow flexed at 90°.

Patients were then instructed to perform, on each side, 6 movements of arm flexion/extension (on the coronal axis), abduction/adduction (on the frontal plane) and internal/external rotation of the arm with adducted elbow.

Each subject was video-recorded from the posterior view, simultaneously to the SHoW Motion task execution and data collection. Male subjects were asked to stand without shirt and female subjects were asked to wear halter top to allow complete observation of the scapulae and posterior thorax.

The scapular angular kinematics were dynamically visualized in three angle–angle plots for each plane of humerus motion (flexion or abduction), in which the three scapular angular motions are plotted against humero-thoracic elevation or abduction (Figs. 2, 3 and following).

**Fig. 1:**
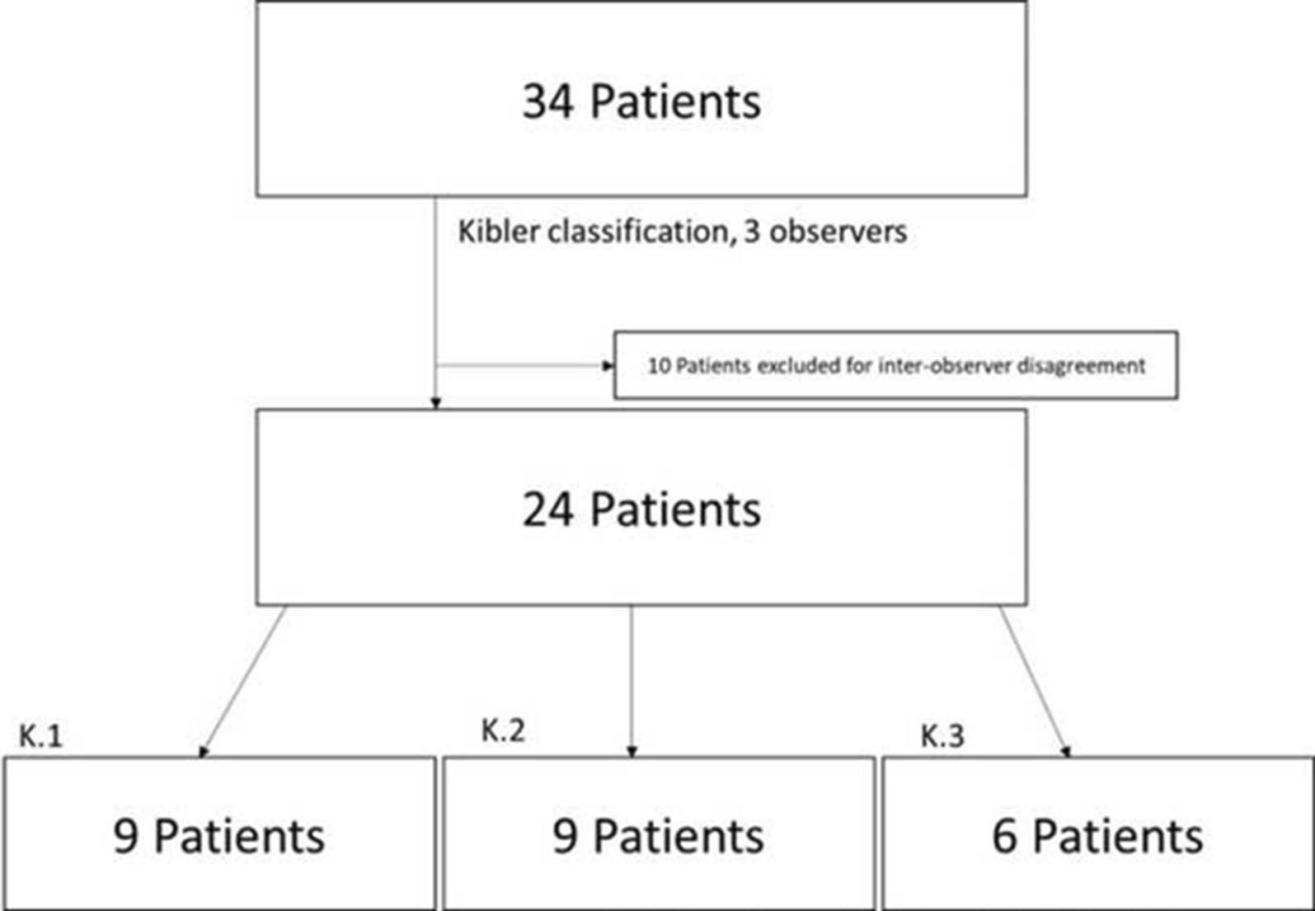
Inclusion criteria and Kibler groups distribution: out of 34 patients, 10 were excluded for inter-observer disagreement. Out of the 24 remaining patients, all the observers agreed on classifying 9 as Kibler 1 (K.1) pattern, 9 as Kibler 2 (K.2) pattern, and 6 as Kibler 3 (K.3) pattern

In a second time three different observers, consisting of a sports medicine resident, a specialized upper arm physiotherapist and an orthopedic resident, viewed each patient’s tape and classified, independently, each shoulder dyskinesis in type I, II or III according to Kibler’s classification. No Kibler IV patients were present, as the described analysis was only performed on patients with SD identified at clinical examination and those patients with contra-lateral healthy shoulder were assigned according to the pattern of the pathological shoulder. Patients on whose classification no unanimous consensus was met within the three observers, were excluded. The remaining patients (24) were then divided into the respective Kibler’s group and, among each of these groups, qualitative plots analysis was performed to identify a commoner pattern (Fig. 1). No control group was added, being the aim of this study to assess the difference within different SD patterns, rather than between SD and non-SD patterns.

## Results

Observing the plots provided by the SHoW Motion 3D kinematic tracking system, a commoner pattern was identified for each Kibler’s class. In particular, a typical pattern was identified within the Kibler I group, another within the Kibler II group and two more, different from each other, within the Kibler III group.

As to better understand the meaning of the alterations described below, normal scapular kinesis is summarized: in sagittal flexion-extension and in lateral abduction-adduction, a progressively increasing scapular tilt, up-ward rotation and retraction is observed [9].

Internal and external rotation plots derived from movements performed with the humerus positioned alongside the body and the elbow flexed at 90° were not deepened in the presented study, as they didn’t provide any useful information.

**Fig. 2:**
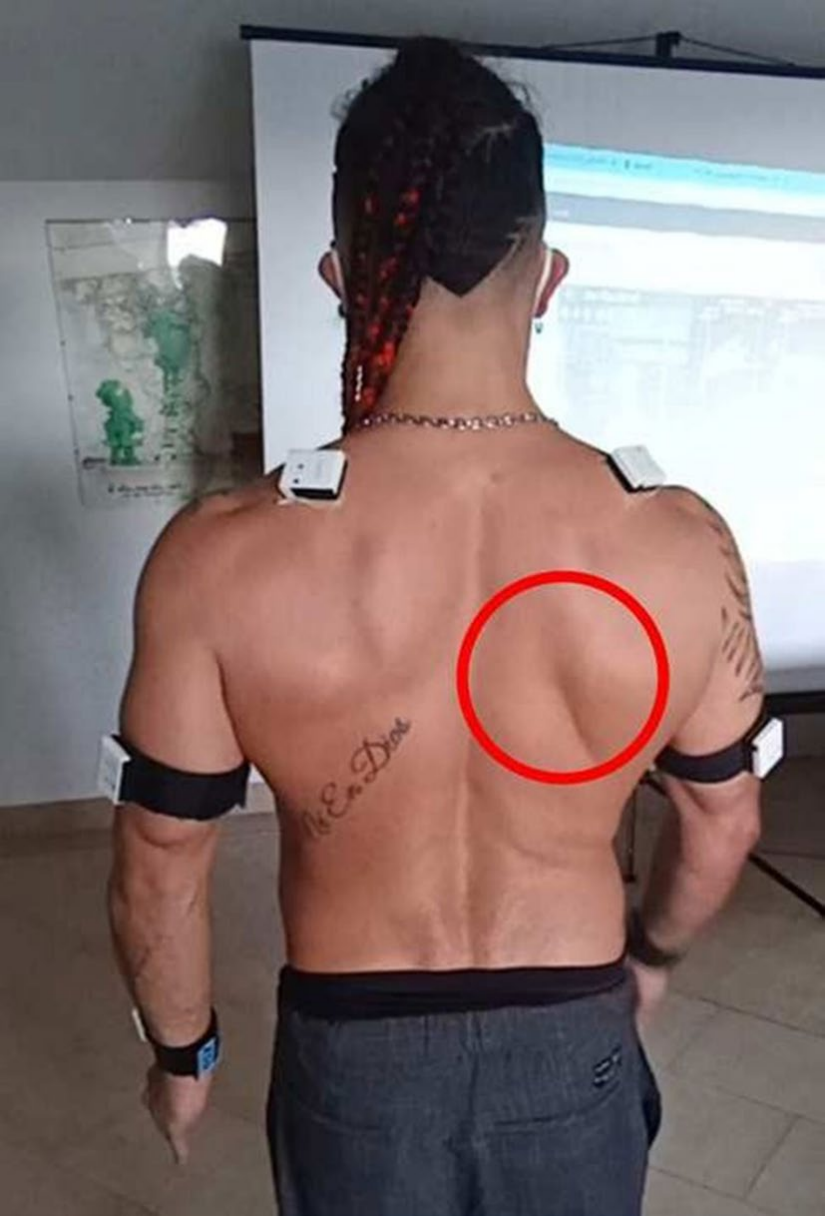
Visual presentation of Kibler type 1, with inferior angle prominence. Dynamically, decreased or reversed posterior tilt and increased protraction in flexion–extension is observed in early degrees of motion

**Fig. 3:**
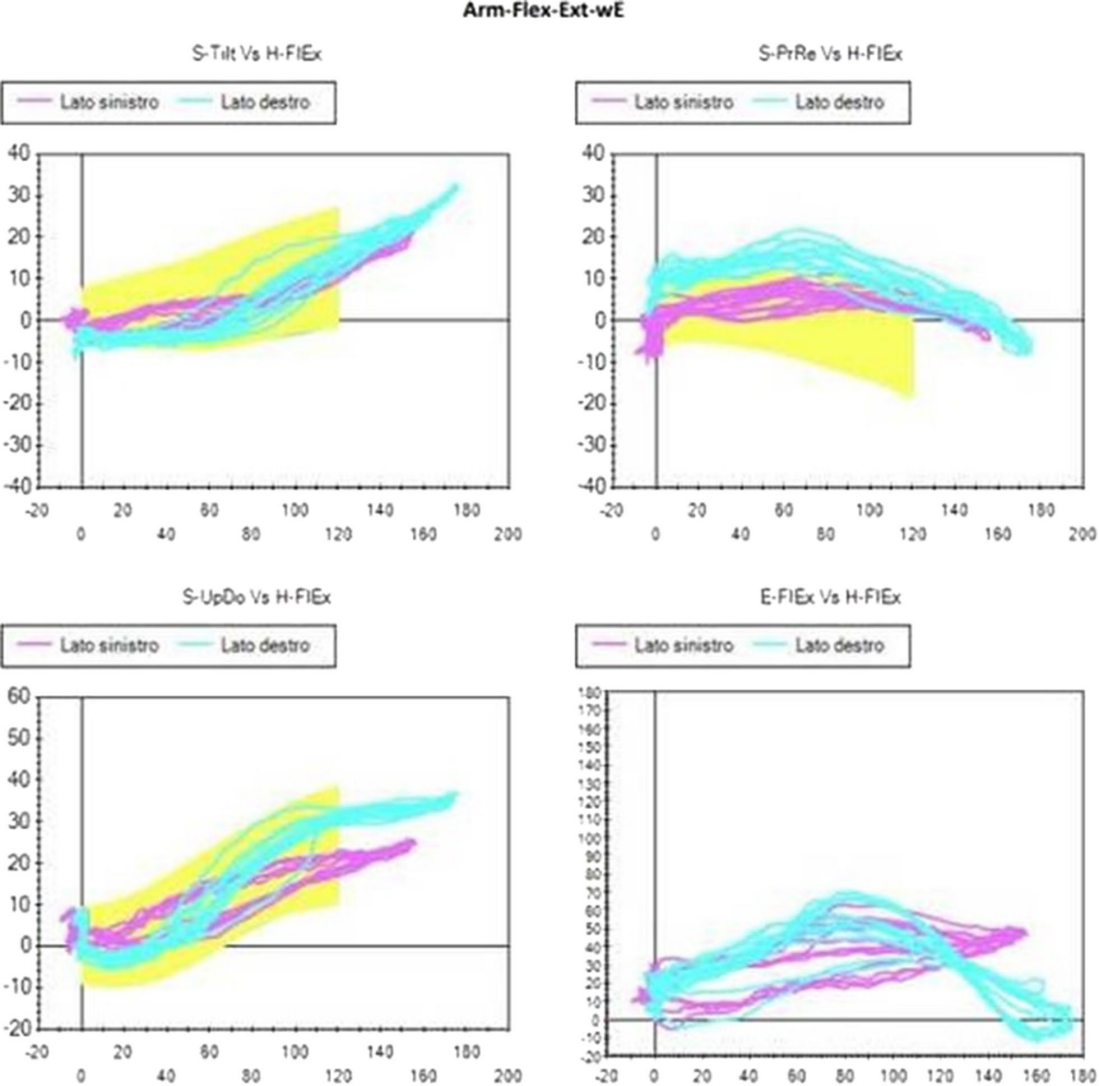
Plot presentation of typical Kibler type 1. In this example, the affected side is the right side, in light blue (left side in purple). Posterior tilt flattening, protraction increase and up-ward rotation reduction are observed with SM analysis, especially in the first 60 degrees of humeral flexion.Upper-left quadrant: posterior tilting (y-axis) with humeral flexion (x-axis). Upper-right quadrant: protraction (y-axis) with humeral flexion (x-axis). Lower-left quadrant: up-ward rotation (y-axis) with humeral flexion (x-axis). Lower-right quadrant: elbow flexion (y-axis) with humeral flexion (x-axis)

Comparing all the Kibler I patients selected as described above, a commoner pattern was observed mainly during the flexion-extension movement. This pattern consisted of a reduction of the posterior tilt, or even in its flattening or inversion, and in an increase of protraction. Slight reduction or inversion of the up-ward rotation during abduction-adduction could be observed in some cases, too. All these characteristics occurred during the first degrees of movement (Figs. 2, 3).

**Fig. 4:**
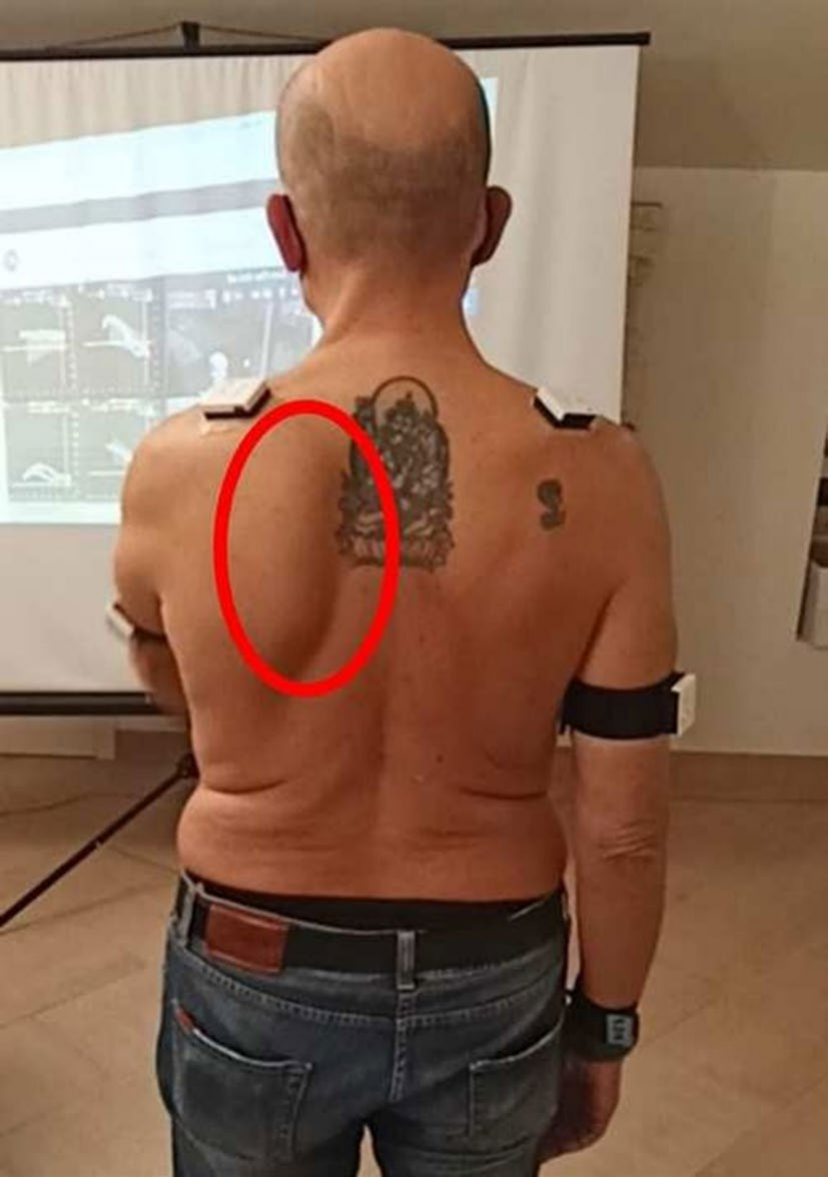
Visual presentation of Kibler type 2, with medial border prominence. Dynamically, increased protraction and marked reversal of lateral rotation in abduction–adduction is observed in early degrees of abduction

**Fig. 5:**
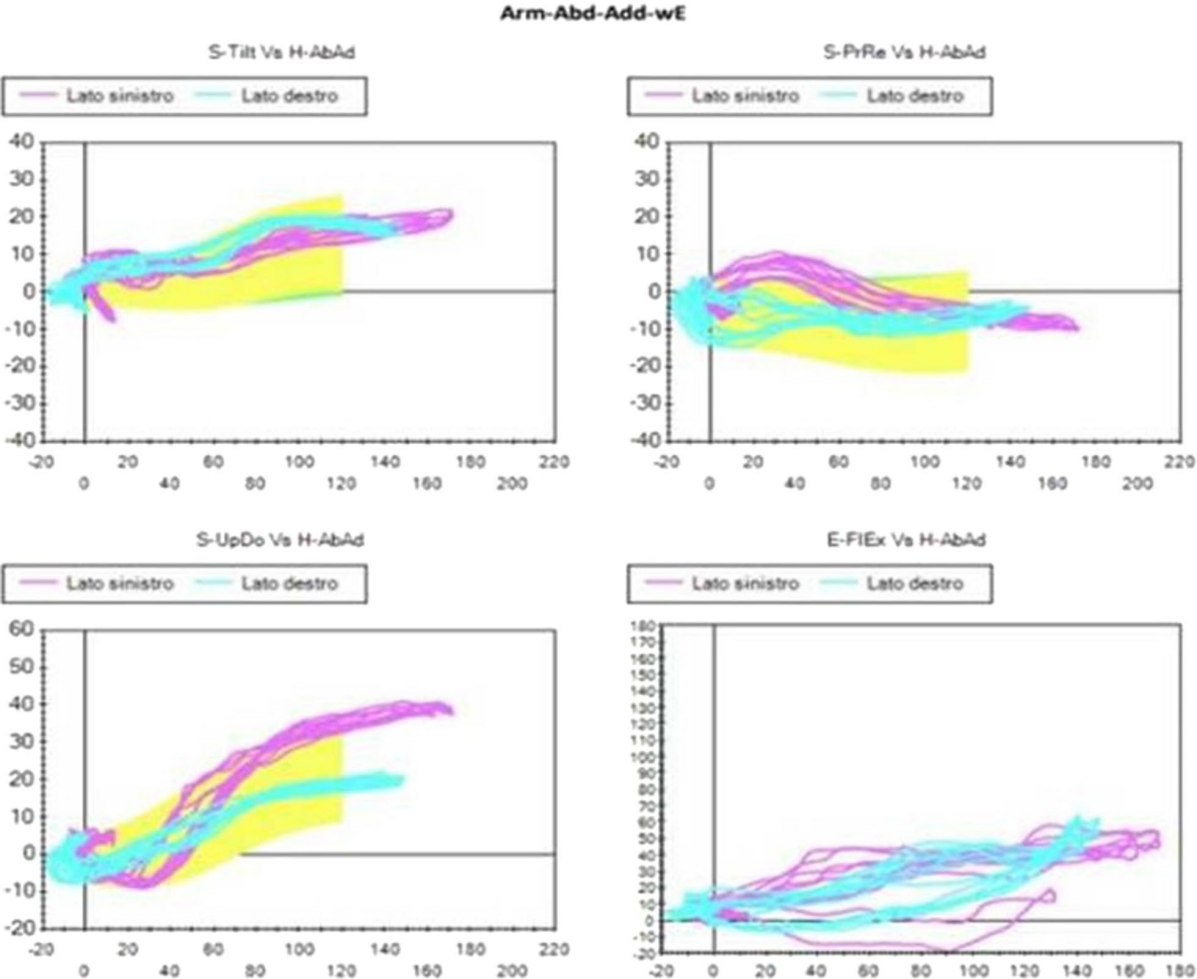
Plot presentation of typical Kibler type 2. In this example, the affected side is the left side, in purple (right side in light blue). Protraction increase and up-ward rotation inversion are observed in the first 60 degrees of abduction, as seen on SM analysis. Upper-left quadrant: posterior tilting (y-axis) with humeral abduction (x-axis). Upper-right quadrant: protraction (y-axis) with humeral abduction (x-axis). Lower-left quadrant: up-ward rotation (y-axis) with humeral abduction (x-axis). Lower-right quadrant: elbow flexion (y-axis) with humeral abduction (x-axis)

Although the Kibler 1 pattern could be observed to some degree in many Kibler 2 patients, and vice versa, a different trend was identified within the group of Kibler 2 patients. This was more evident in the abduction-adduction and consists in an increase in protraction and in a marked inversion of the up-ward rotation in the first degrees of movement. A smaller inversion of the up- ward rotation in the first degrees of movement could be observed during flexion-extension, too. Therefore, although similar compared with Kibler pattern 1, Kibler pattern 2 is distinguished by a pronounced protraction in abduction-adduction (Figs. 4, 5).

The Kibler III group was divided into 2 sub-groups, one composed of patients with massive rotator cuff tear who presented the “shrug” movement of the scapula, and one composed of patients with capsular stiffness and/or impingement limiting the last degrees of arm elevation.

**Fig. 6:**
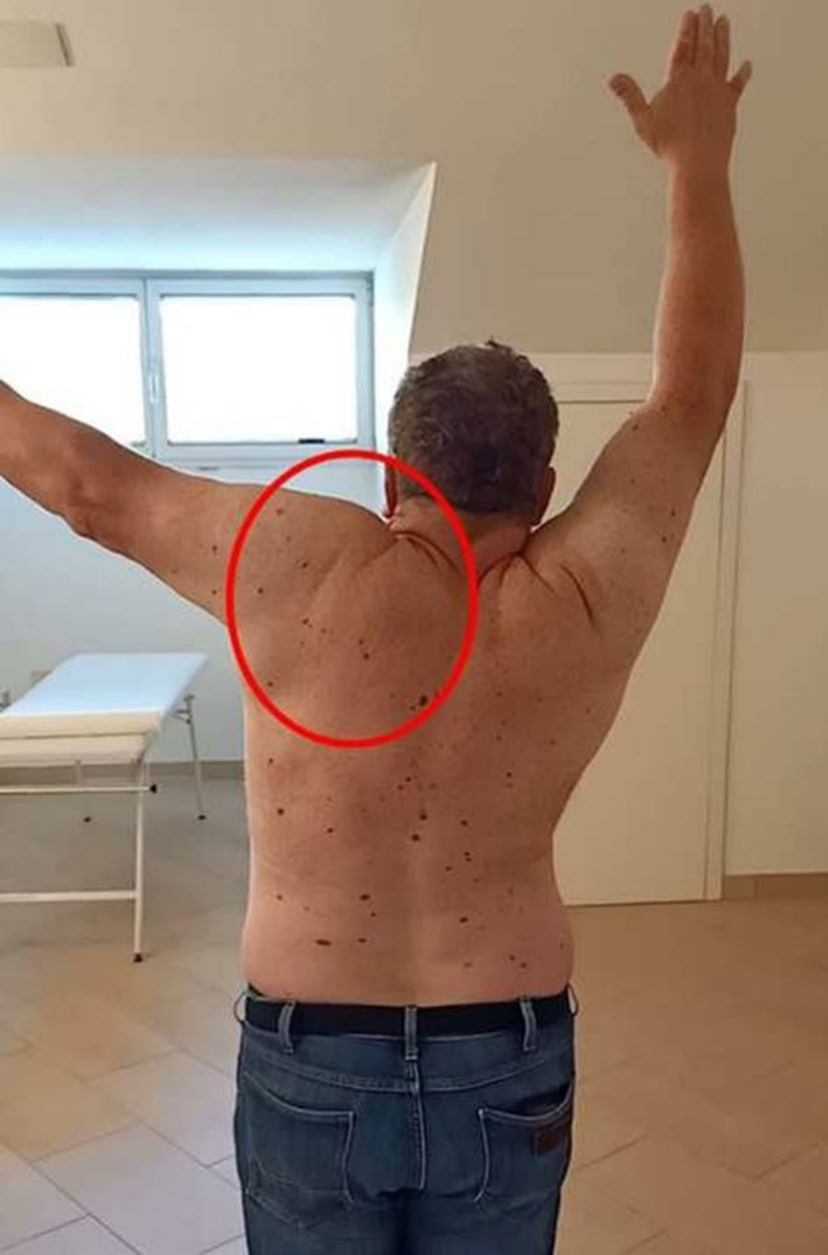
Visual presentation of Kibler type 3 (A), with a “shrug” movement characterized by an increase of scapular elevation

**Fig. 7:**
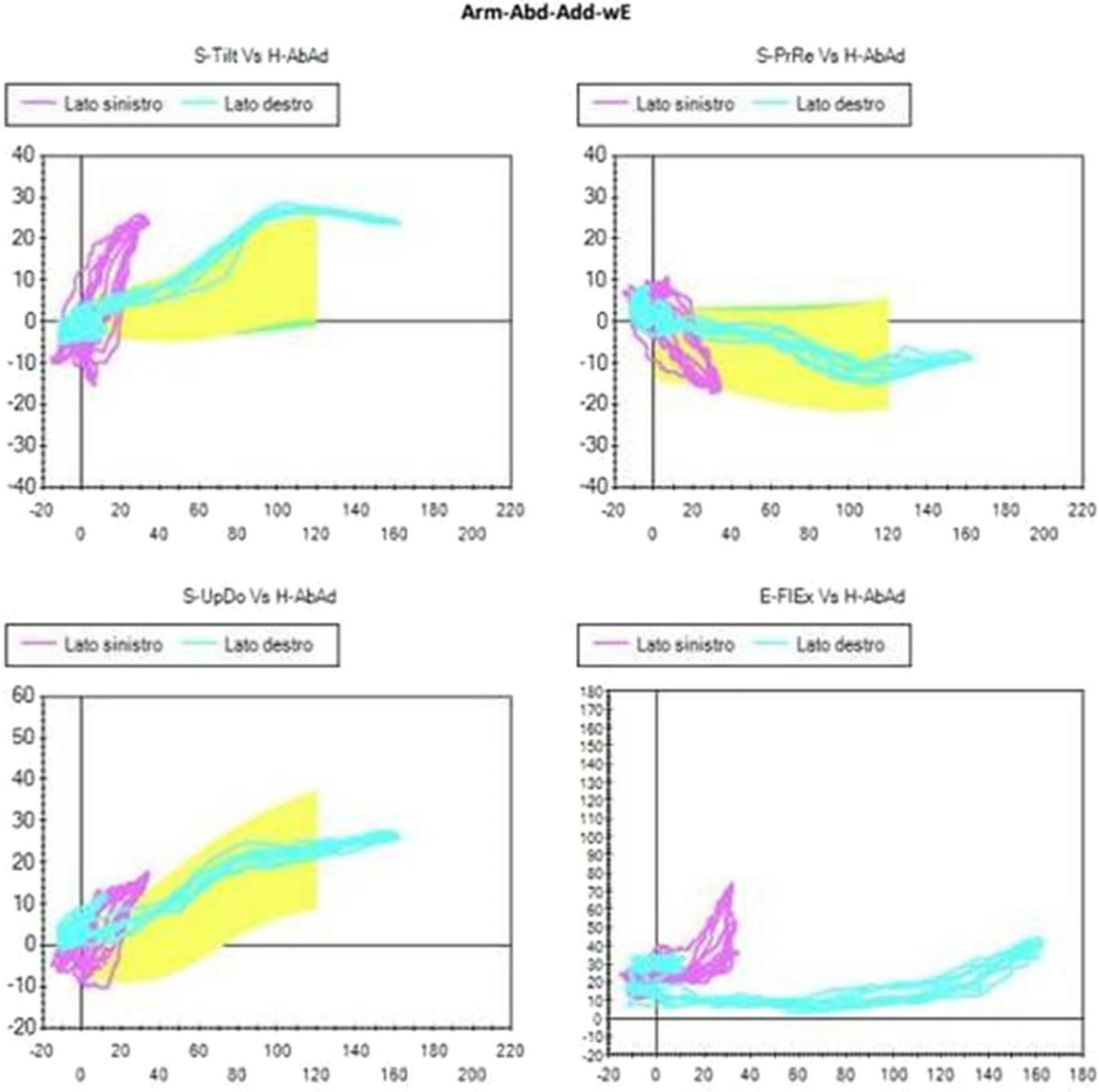
Plot presentation of typical Kibler type 3(A). In this example, the affected side is the left side, in purple (right side in light blue). An increase in all scapular movement angles starting from the beginning of the movement is observed in abduction, as shown on the SM analysis. Upper-left quadrant: posterior tilting (y-axis) with humeral abduction (x-axis). Upper-right quadrant: protraction (y-axis) with humeral abduction (x-axis). Lower-left quadrant: up-ward rotation (y-axis) with humeral abduction (x-axis). Lower-right quadrant: elbow flexion (y-axis) with humeral abduction (x-axis)

The first sub-group was named Kibler III-A and presented, in both flexion-extension and abduction-adduction, an increase in all scapular movement angles starting from the beginning of the movement, which was also markedly restricted (Figs. 6, 7)

**Fig. 8:**
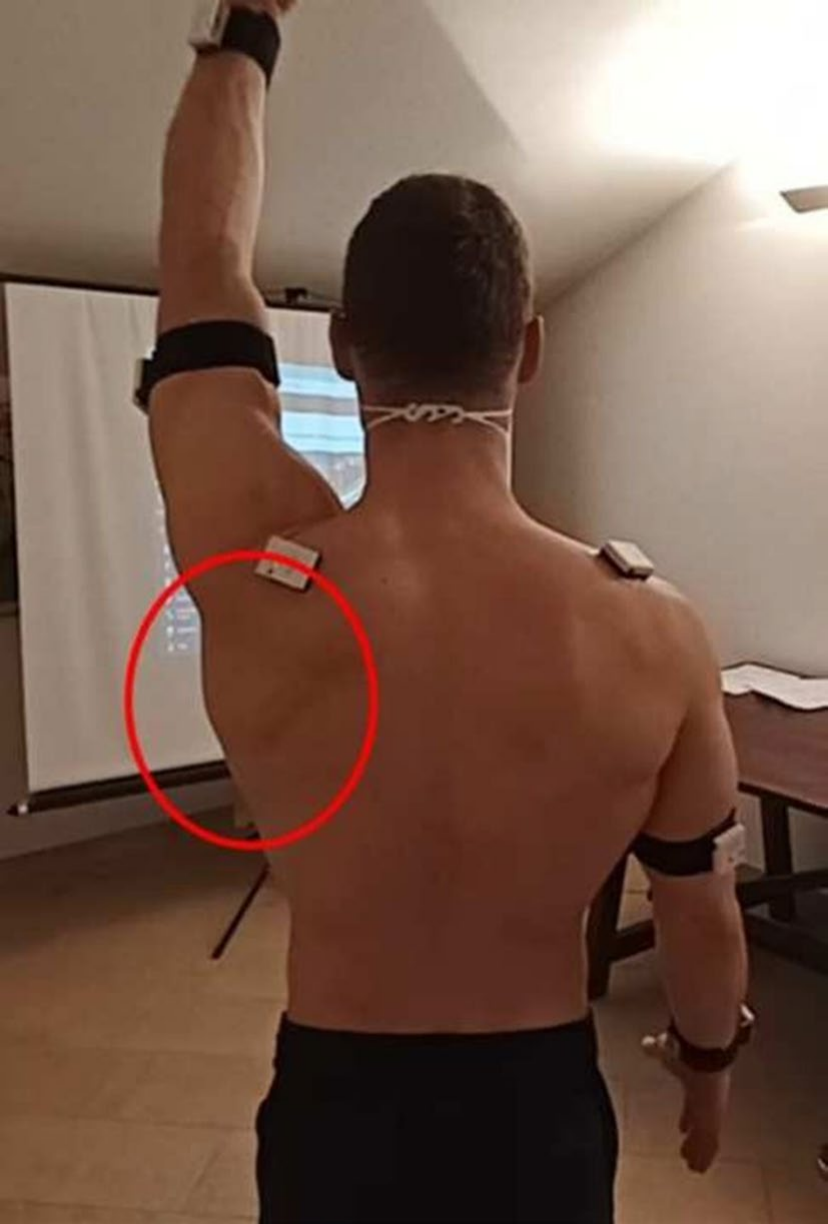
Visual presentation of Kibler type 3 (B), with dynamical superior border elevation consequent to increased scapular up-ward rotation

**Fig. 9:**
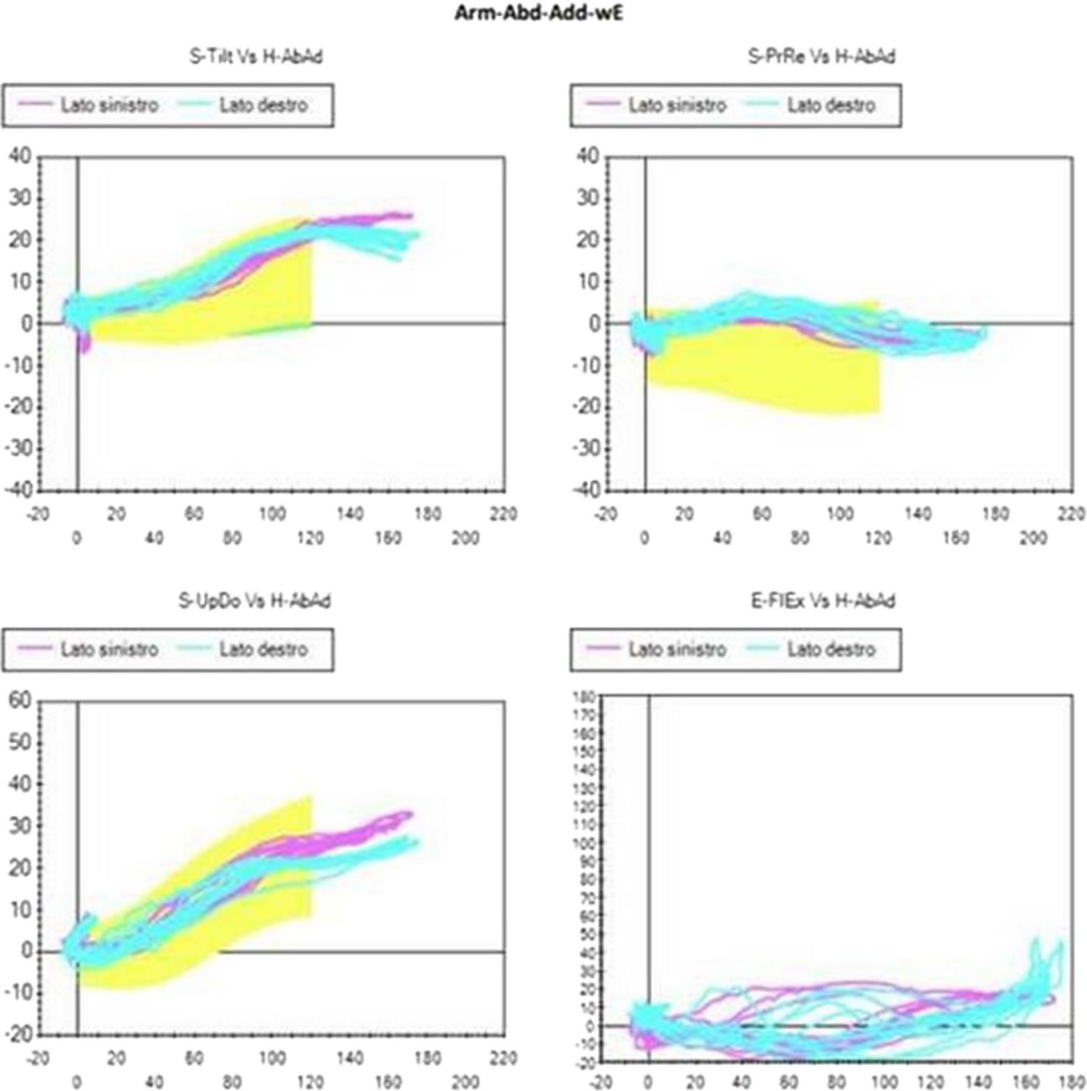
Plot presentation of typical Kibler type 3(B), In this example, the affected side is the left side, in purple (right side in light blue). Posterior tilting and up-ward rotation increase is observed at the final degrees (over 100) of abduction, as shown on the SM analysis. Upper-left quadrant: posterior tilting (y-axis) with humeral abduction (x-axis). Upper-right quadrant: protraction (y-axis) with humeral abduction (x-axis). Lower-left quadrant: up-ward rotation (y-axis) with humeral abduction (x-axis). Lower-right quadrant: elbow flexion (y-axis) with humeral abduction (x-axis)

The second sub-group was named Kibler III-B and was characterized by a slight increase in posterior tilting and up-ward rotation at the final degrees of flexion-extension and abduction- adduction (Figs.8, 9).

It should be noted that the described trends were usually slightly more evident on the pathological side, but were almost always observed bilaterally, so that they were identified by performing a comparison with the average of the normal population, rather than with the contralateral arm. This comparison was made taking into account the validated reference values for the used sensors [9], which globally consist of minimal posterior tilt, up-ward rotation and retraction in the first 30 degrees of humeral flexion and abduction, all increasing in subsequent degrees of movement.

## Discussion

Appropiate 3D measurment of the scapular kinematics during dynamic movement provides additional means to make an accurate diagnosis and, thus, set the correct treatment. A proper movement analysis, depicted on accurate plots, can be compared to an extremely detailed picture on the functional status of the patient. As already demonstrated [10], the SHoW Motion 3D kinematic tracking system allows an extremely low intra and infra-operator variability, making possible the achievement of follow-ups with never seen before information on the functional improvement of the patient.

There are several methods for assessing SD, but many are performed in the static position, albeit well accurate [11], or in dynamic position but without sufficient accuracy on the scapular movement. We believe that the equipment used in the study meets all the requirements for a proper SD evaluation. A study similar to this, and conducted by the same research group, already proved the SM to be a paramount method to assess scapulohumeral rhythm in shoulders with reverse shoulder arthroplasty [12].

The Kibler type 1 pattern is characterized by the protrusion of the inferior angle of the scapula. As observed in our analysis, this is a consequence of a reduction in posterior tilt, or even an anterior tilt stance of the scapula, in addition to a scapular protraction. Besides this, because of the weakness of the periscapular musculature, the upward rotation that accompanies humeral elevation in the first degrees is lacking. However, this last pattern is more typical of Kibler type II where muscular weakness predominates especially in abduction-adduction. In the Kibler type I group, instead, SD was observed mainly during the flexion-extension movement, when the arm "weighs" on the scapula and induces an anterior tilt and protraction which is not adequately balanced, while the scapula is better stabilized in the abduction-adduction, which is not much different from the scaption movement. There is therefore a certain overlap between Kibler pattern I and II, but overall in the first group the SD is more objectifiable in flexion-extension. The Kibler pattern type II, however, is more identifiable in abduction-adduction because this movement recruits muscles whose weakness does not allow an adequate control of the up-ward rotation.

Kibler pattern type 3 has been divided into two subgroups, A and B. Type A is typical of patients with an extensive cuff lesion in whom the function of the glenohumeral joint is severely compromised and is therefore characterized by a marked increase in all scapular movement angles, as a consequence of a greater use of the scapulothoracic joint, which replaces the non- functioning glenohumeral joint. Type B, on the other hand, belongs to patients with cuff stiffness or impingement that limits the final degrees of humeral elevation. What is observed is that, in correspondence of these degrees of flexion and abduction, there is an increase in posterior tilt and up-ward rotation in order to compensate the block of the glenohumeral joint.

In conclusion, our study finally breaks the main limit of Kibler’s classification, which is inter and intra operator variability. This type of analysis puts the recorded movement “down on paper”, through precise graphs, and goes beyond the need of the operator's interpretation. In other words, we have made Kibler's classification no longer an opinion but an analytical evaluation.

The method we present allows a more accurate classification than the simple Kibler one, in a dynamic, safe, non-invasive, fast, reproducible, accurate, not excessively expensive and definitely cost-effective way. Moreover, it provides an objective evaluation of the motor pattern alteration, which is an essential tool in follow-up in order to evaluate the effectiveness of the conservative or surgical treatment.
